# MAPK4 facilitates angiogenesis by inhibiting the ERK pathway in non‐small cell lung cancer

**DOI:** 10.1002/cai2.117

**Published:** 2024-04-16

**Authors:** Jing Chen, Jing Yang, Yufang Liu, Xu Zhao, Juanjuan Zhao, Lin Tang, Mengmeng Guo, Ya Zhou, Chao Chen, Dongmei Li, Zhenke Wen, Guiyou Liang, Lin Xu

**Affiliations:** ^1^ Special Key Laboratory of Gene Detection & Therapy of Guizhou Province Zunyi Guizhou China; ^2^ Department of Immunology Zunyi Medical University Zunyi Guizhou China; ^3^ Department of Medical Physics Zunyi Medical University Zunyi Guizhou China; ^4^ Institute of Biomedical Soochow University Suzhou Jiangsu China; ^5^ Department of Cardiovascular Surgery Affiliated Hospital of Guizhou Medical University Guiyang Guizhou China

**Keywords:** angiogenesis, endothelial cell, MAPK4, NSCLC, p‐ERK1/2

## Abstract

**Background:**

Angiogenesis plays an important role in the occurrence and development of non‐small cell lung cancer (NSCLC). The atypical mitogen‐activated protein kinase 4 (MAPK4) has been shown to be involved in the pathogenesis of various diseases. However, the potential role of MAPK4 in the tumor angiogenesis of NSCLC remains unclear.

**Methods:**

Adult male C57BL/6 wild‐type mice were randomly divided into the control group and p‐siMAPK4 intervention group, respectively. The cell proliferation was analyzed with flow cytometry and immunofluorescence staining. The vascular density in tumor mass was analyzed by immunofluorescence staining. The expressions of MAPK4 and related signaling molecules were detected by western blot analysis and immunofluorescence staining, and so on.

**Results:**

We found that the expression of MAPK4, which was dominantly expressed in local endothelial cells (ECs), was correlated with tumor angiogenesis of NSCLC. Furthermore, MAPK4 silencing inhibited the proliferation and migration abilities of human umbilical vein ECs (HUVECs). Global gene analysis showed that MAPK4 silencing altered the expression of multiple genes related to cell cycle and angiogenesis pathways, and that MAPK4 silencing increased transduction of the extracellular regulated protein kinases 1/2 (ERK1/2) pathway but not Akt and c‐Jun n‐terminal kinase pathways. Further analysis showed that MAPK4 silencing inhibited the proliferation and migration abilities of HUVECs cultured in tumor cell supernatant, which was accompanied with increased transduction of the ERK1/2 pathway. Clinical data analysis suggested that the higher expression of MAPK4 and CD34 were associated with poor prognosis of patients with NSCLC. Targeted silencing of MAPK4 in ECs using small interfering RNA driven by the CD34 promoter effectively inhibited tumor angiogenesis and growth of NSCLC in vivo.

**Conclusion:**

Our results reveal that MAPK4 plays an important role in the angiogenesis and development of NSCLC. MAPK4 may thus represent a new target for NSCLC.

## Graphical abstract

(a) MAPK4 facilitates tumor angiogenesis and progression in NSCLC by promoting the growth of endothelial cell (CD34^+^ EC), which is related to altered transduction of Raf/MEK/ERK1/2 signaling pathway. (b) Targeted intervention of the expression of MAPK4 in CD34+ ECs can repress tumor angiogenesis and progression of NSCLC.

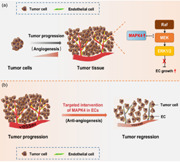

**Abbreviations**
AKT: protein kinase B
ECs: endothelial cells
ERK1/2: extracellular regulated protein kinases 1/2
FCM: flow cytometry
HUVECs: human umbilical vein endothelial cells
JNK: c‐Jun n‐terminal kinase
Ki67: cell proliferation nuclear antigen
LLC: Lewis lung carcinoma
MAPK4: mitogen‐activated protein kinase 4
MEK: mitogen‐activated extracellular signal‐regulated kinase
NF‐κB: nuclear factor κB
NSCLC: non‐small cell lung cancer
p21: cyclin‐dependent kinase inhibitor 1A
p‐AKT: phosphorylated AKT
p‐ERK1/2: phosphorylated ERK1/2
p‐JNK: phosphorylated JNK
p‐MEK: phosphorylated MEK
p‐NF‐κB: phosphorylated NF‐κB
Raf: rapidly accelerated fibrosarcoma
Ras: rat sarcoma
RNAi: RNA interference
siRNA: small interfering RNA
WT: wild‐type

## 1 INTRODUCTION

Non‐small cell lung cancer (NSCLC) is a highly morbid and lethal disease with a poor prognosis that accounts for 85% of all lung cancers [1, 2]. Approximately 70% of patients with newly diagnosed NSCLC are over 65 years of age and more than half have metastatic or locally advanced NSCLC [3]. Despite recent advances in the diagnosis and treatment of NSCLC, the 5‐year overall survival rate remains low [4], especially in the elderly population. The low survival rate is in part because of the complex etiology of NSCLC tumorigenesis. Research has shown that angiogenesis plays an important role in the occurrence and development of NSCLC. Angiogenesis is necessary for tumor cells to further grow after penetrating the epithelial basement membrane and has important clinical significance for tumor growth and metastasis and even prognosis [5, 6]. For example, Zhang et al. [7] found that high expression of ubiquitin‐specific protease 22 enhanced angiogenesis and recurrence of NSCLC. Zhuo et al. [8] found that cadherin‐2 induced angiogenesis, thus promoting the metastasis of lung adenocarcinoma. The growth and migration of endothelial cells (ECs) are the key factors in mediating neovascularization [9, 10]. Therefore, angiogenesis inhibitors, such as bevacizumab, ramucirumab, and nintedanib, have been proposed as clinical antitumor drugs [11, 12]. Although antiangiogenic therapies have been extensively evaluated in patients with NSCLC, these antiangiogenic agents have showed no significant positive effects on all stages of NSCLC. Therefore, it is of great significance to further study the antiangiogenesis mechanisms in NSCLC, especially the molecular mechanism underlying the growth and migration of ECs. Additionally, the combination of antiangiogenic agents with other therapies, such as gene therapy [13] and traditional Chinese medicine [14], should be investigated, as these combinations may have a better clinical therapeutic effect than any single therapeutic agent [15, 16].

Mitogen‐activated protein kinase 4 (MAPK4; also called ERK4 or p63MAPK), a 578 aa protein with a molecular weight of ~70 kDa, is a member of the atypical MAPK family [17]. Accumulating evidence has shown that MAPK4 is an important intrinsic regulatory factor in the development of various organs and diseases. For example, our recent work showed that MAPK4 is involved in the pathological changes of acute lung injury (ALI) [18] and autoimmune hepatitis [19]. Recent studies also demonstrated that MAPK4 is also closely related to the development of multiple cancers [20, 21, 22]. For example, MAPK4 has been reported to modulate the immune response in cancers and mediate tumorigenesis in cancers such as cervical cancer [23], ovarian cancer [24], gastric cancer [25], and breast cancer [26]. Moreover, MAPK4 was shown to promote the growth of human NSCLC cell lines [27], indicating that it might play an important role in the occurrence and development of NSCLC. Therefore, it is of significance to explore the potential role of MAPK4 in angiogenesis in tumors, including NSCLC, which would be much valuable for the development of new clinical strategies against tumors.

In the present study, we assessed the potential role of MAPK4 in angiogenesis of NSCLC using a murine NSCLC tumor model. Our data showed that the expression of MAPK4 was dominant in ECs of tumor tissue, accompanied by an increase in local angiogenesis of NSCLC. MAPK4 silencing inhibited the proliferation and migration abilities of human umbilical vein ECs (HUVECs), accompanied with increased transactivation of extracellular regulated protein kinases 1/2 (ERK1/2) pathway. Analysis of clinical data indicated that the high expression of both MAPK4 and CD34 in tumor tissues were associated with poor prognosis of patients with NSCLC. Finally, targeted intervention of MAPK4 expression in ECs not only affected angiogenesis but also markedly inhibited the growth of NSCLC in vivo. Collectively, our findings reveal a previously unknown role of MAPK4 in the angiogenesis of NSCLC and indicate that targeted intervention of the expression of MAPK4 in ECs may be a viable clinical antiangiogenic therapeutic strategy for NSCLC treatment.

## 2 MATERIALS AND METHODS

### 2.1 Animals

C57BL/6 wild‐type (WT) mice (5–6 weeks old) provided by Zunyi Medical University Laboratory Animal Care were housed under specific pathogen‐free conditions at Zunyi Medical University. All animal experiments were performed following the Guidelines for the Care and Use of Laboratory Animals (Ministry of Health, China, 1998). The experimental procedures were approved by the ethical guidelines of the Zunyi Medical University Laboratory Animal Care and Use Committee (permit number 2018016).

### 2.2 Cell culture

Lewis lung carcinoma (LLC) cells were cultured in high‐glucose Dulbecco's modified Eagle medium containing 10% fetal bovine serum at 37°C in 5% CO_2_. HUVECs and A549 cells were cultured in RPMI 1640 medium containing 10% fetal bovine serum at 37°C in 5% CO_2_. HUVECs (Cellosaurus accession ID: CVCL_CVCL_B7UI) and A549 (CVCL_0023) cells were purchased from the Conservation Genetics CAS Kunming Cell Bank (KCB200603YJ) and short tandem repeat‐profiled at the Conservation Genetics CAS Kunming Cell Bank. All experiments were performed with mycoplasma‐free cells.

### 2.3 Establishment of the NSCLC model

Adult male C57BL/6 mice were randomly divided into the control group and p‐siMAPK4 intervention group, respectively. To establish the mouse NSCLC tumor model, the mice were implanted with the LLC NSCLC cell line (5 × 10^5^ tumor cells/mouse).

### 2.4 Immunofluorescence

Frozen sections of tumor tissues were obtained and sliced into 6‐µm‐thick sections. After deparaffinization and rehydration, the slides were incubated with the corresponding primary antibodies (anti‐MAPK4: Proteintech, 26102‐1‐AP; anti‐phosphorylated ERK1/2 [p‐ERK1/2]: Abcam, ab201015; anti‐CD34: Abcam, ab8158; anti‐cell proliferation nuclear antigen [Ki67; Abcam, 92742]) and secondary antibodies (Alexa Fluor 647: Cell Signaling, 4414S; Alexa Fluor 488: Abcam, ab150077; anti‐MAPK6: Absin, abs133606; anti‐NLK: Abcam, ab26050; anti‐MAPK15: Thermo Fisher, PA5‐105740). The slides were then counterstained with 4′,6‐diamidino‐2‐phenylindole (Beyotime, C1002) and observed under an Olympus microscope.

### 2.5 RNA extraction, reverse transcription, and quantitative real‐time polymerase chain reaction (PCR)

Total RNA was isolated from HUVECs using RNAiso Plus (TAKARA, 9108) following the manufacturer's instructions. RNA was quantified and reverse transcribed to complementary DNA (cDNA) with the PrimeScript 1st Strand cDNA Synthesis kit SuperMix for qPCR (TAKARA, 6110A) and following the manufacturer's instructions. Real‐time PCR amplifications were performed on the Bio‐Rad CFX96 detection system (Bio‐Rad Laboratories) in 20 μL reaction volumes containing cDNA, primers, and TB Green® Premix Ex Taq^TM^ II (Tli RNaseH Plus) (TAKARA, RR820A). The relative expression levels of genes were calculated by the comparative threshold cycle method with glyceraldehyde 3‐phosphate dehydrogenas (GAPDH) messenger RNA (mRNA) as the internal reference.

### 2.6 Flow cytometry (FCM)

The expression of Ki‐67 (Ki67‐Alexa Fluor®488 antibodies, 350507, BioLegend) and the cell cycle distribution of cells were detected by FCM with a Beckman Gallios flow cytometer (Beckman Coulter, Inc.). Intracellular staining was conducted following the manufacturer's manual (eBioscience, Fixation/Permeabilization kit, 00‐5123‐43).

### 2.7 Western blot analysis

HUVECs were homogenized in ice‐cold lysis buffer (KeyGEN BioTECH, KGP2100) following the manufacturer's instructions. Equal amounts of protein were separated by 10% sodium dodecyl‐sulfate polyacrylamide gel electrophoresis and transferred onto polyvinylidene difluoride membranes. Membranes were incubated with 5% skim milk in phosphate‐buffered saline (PBS) for 1 h. Immunoblotting was performed using monoclonal antibodies against cyclin‐dependent kinase inhibitor 1A (p21) (Proteintech, 10355‐1‐AP), Cyclin A (Abcam, ab185619), Cyclin B1 (Abcam, ab181593), MAPK4 (Proteintech, 26102‐1‐AP), protein kinase B (AKT) (Abcam, ab8805), phosphorylated AKT (p‐AKT) (Abcam, ab38449), ERK1/2 (Abcam, ab184699), p‐ERK1/2 (Abcam, ab201015), nuclear factor κB (NF‐κB) (Abcam, ab32536), phosphorylated NF‐κB (p‐NF‐κB) (Abcam, 97726), c‐Jun n‐terminal kinase (JNK) (Abcam, ab179461), phosphorylated JNK (p‐JNK) (Abcam, ab124956), rat sarcoma (Ras) (Abcam, ab52939), rapidly accelerated fibrosarcoma (Raf) (Abcam, ab137435), p‐Raf (Abcam, ab173539), mitogen‐activated extracellular signal‐regulated kinase (MEK) (Abcam, ab178876), phosphorylated MEK (p‐MEK) (Abcam, ab194754), and GAPDH (Abcam, 181602). Membranes were washed in PBS with Tween 20 and subsequently incubated with horseradish peroxidase‐conjugated anti‐rabbit secondary antibody (Abcam, 7074S). Signals were detected and analyzed using a Bio‐Rad ChemiDoc MP Imaging System (Bio‐Rad Laboratories). GAPDH was used as the internal reference.

### 2.8 Plasmid construction

A series of CD34 promoter truncations (UCSC, ENSMUST00000016638.7) and the MAPK4 small interfering RNA (siRNA) construct were synthesized and cloned into the pGL3.0 basic vector between the SacI and XhoI sites (Gene Create, GS1‐1905109). The vectors were purified using an EndoFree Plasmid Maxi Kit (12123) and used for experiments after verification by DNA sequencing.

### 2.9 Plasmid transfection in vivo

Plasmid (75 µg) and Entranster^TM^‐in vivo (Engreen, 18668‐11‐2) (150 µL) were mixed at a ratio of 1:2 used to form the in vivo transfection complex. After incubation at room temperature for 15 min, the transfection complex (containing 75 µg plasmid) was injected into the distal portion of the tumor once every 2 days for a total of three times.

### 2.10 Hematoxylin‐eosin (H&E) staining

Tumor and lung tissues from the mouse xenograft tumor model were subjected to paraffin embedding, sectioning, and HE staining with the assistance of the Department of Pathology, Affiliated Hospital of Zunyi Medical University.

### 2.11 siRNA tranfection

HUVECs were inoculated into 24‐well plates and cultured in RPMI 1640 medium containing 10% fetal bovine serum at 37°C in 5% CO_2_. When cells reached 70%–80% confluence, the cells were transfected with MAPK4 siRNA (50 nM) using Lipofectamine 3000 reagent (Invitrogen, L3000015) following the manufacturer's instructions.

### 2.12 Cell counting kit‐8 (CCK‐8) assay

CCK‐8 reagent (MCE, HY‐K0301) in fresh complete medium (1: 9) was added to transfected HUVECs seeded in 96‐well plates and incubated for 2 h. The optical density at 450 nm (OD_450_) in each well was measured in the dark with a microplate reader, and the absorbance curve was generated.

### 2.13 Colony formation assay

Transfected HUVECs were seeded in six‐well plates at 50 or 100 cells per well for different experimental setting, respectively. After 14 days of culture, 4% paraformaldehyde was added for 15 min and 0.5% crystal violet staining solution was then added for 20 min. The plates were washed and the colonies were counted and photographed.

### 2.14 Tube formation assay

Matrigel basement membrane matrix (BD, 356234) was used to coat 96‐well plates (60 µL per well). After the Matrigel solidified, HUVECs were seeded into the wells (2 × 10^4^ cells per well) and cultured in serum‐free complete medium for 12 h. Tube formation was observed under a microscope (Olympus IX‐51 inverted optical microscope).

### 2.15 Patients and tissue samples

Lung cancer paraffin samples were obtained from Shanghai Outdo Biotech Company (HlugA180Su03). This study was approved by the Ethics Committee of the Shanghai Outdo Biotech Company.

### 2.16 Statistical analysis

Statistical analysis was performed using GraphPad Prism 8 software (GraphPad Prism 8.0.1). One‐way analysis of variance followed by Bonferroni's post hoc test was applied for multiple comparisons, and Student's *t* test was used when two conditions were compared. A two‐tailed *p* < 0.05 was considered statistically significant. All data are shown as the mean ± SEM values. Survival was evaluated by the Kaplan–Meier method.

## 3 RESULTS

### 3.1 MAPK4 expression and angiogenesis of NSCLC

**Figure 1:**
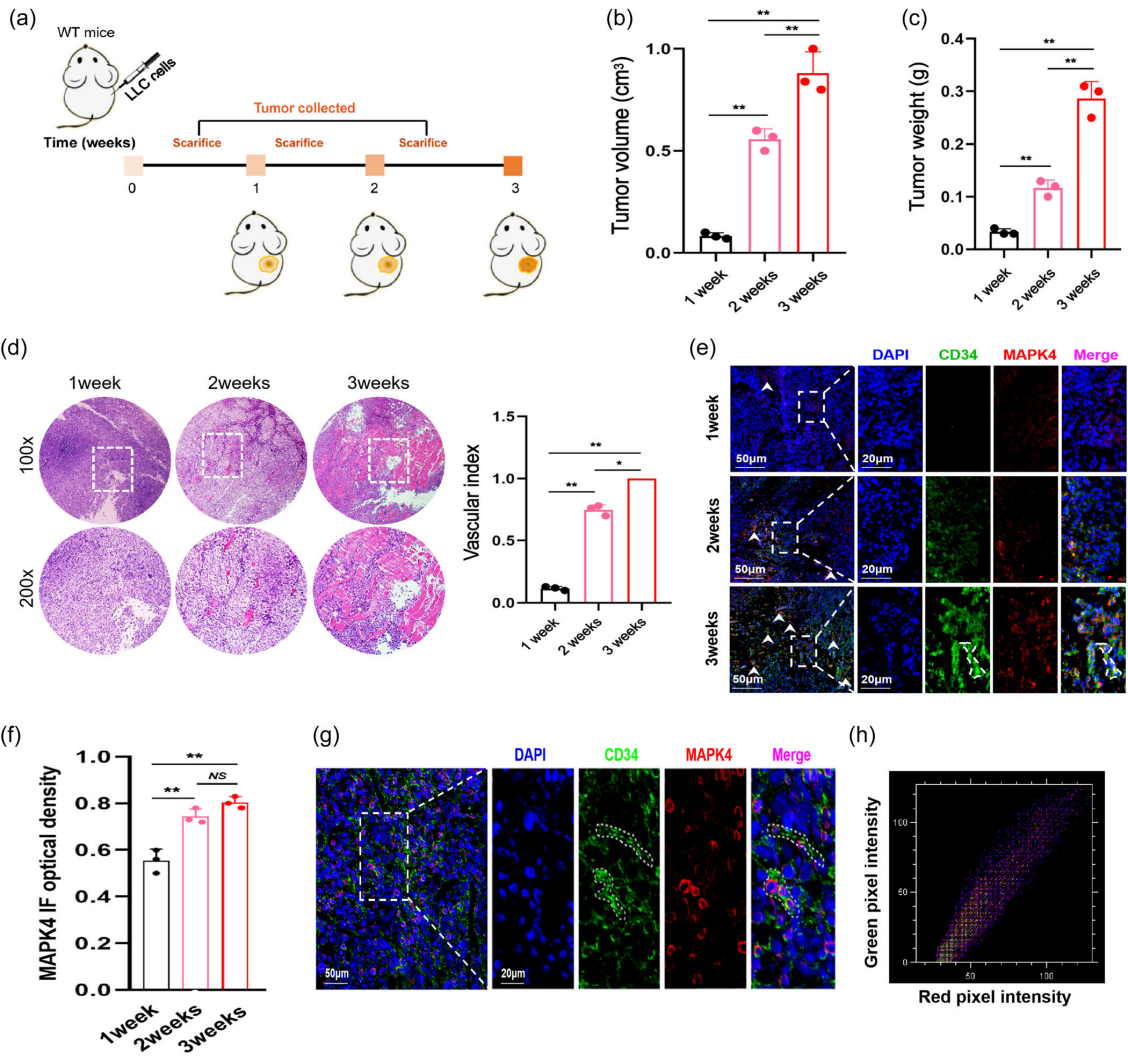
Mitogen‐activated protein kinase 4 (MAPK4) expression during the development of non‐small cell lung cancer (NSCLC). (a) Schematic diagram showing subcutaneous injection of Lewis lung carcinoma (LLC) cells (5 × 10^5^) into the right flanks of wild‐type (WT) mice. (b) Tumor volume. (c) Tumor weight. (d) Tumor pathology was analyzed by hematoxylin‐eosin (H&E) staining. (e) The expression levels of MAPK4 in tumors was analyzed by immunofluorescence and (f) quantified. (g) The colocalization of CD34 and MAPK4 in tumors were analyzed by immunofluorescence. (h) Colocation coefficient between CD34 and MAPK4 expression were quantified by ImageJ. Representative data from three independent experiments were shown. *p < 0.05, **p < 0.01. NS, no significance.

To explore the potential relationship between MAPK4 and angiogenesis during the development of NSCLC, we examined the expression of atypical MAPK family members at indicated time points after establishment of the NSCLC tumor model in WT mice (Figure 1a). The tumor volume and weight markedly increased over time in the NSCLC model mice (Figure 1b,c) Furthermore, the expression level of CD34, a marker of ECs, in tumor tissues increased, indicating the increase of local vascular density during the tumorigenesis of NSCLC (Figure 1d). Further analysis showed that the expression level of MAPK4 was elevated in CD34^+^ ECs in tumor tissues (Figure 1e,f), but no changes were observed for the other atypical MAPK family members, such as MAPK6, NLK, and MAPK15 (Supporting Information S1: Figure S1A–D). These results indicated a positive correlation between MAPK4 and vascular density during the progression of NSCLC. We further found that MAPK4 and CD34 strongly colocalized in tumors (Figure 1g,h). Together, these data showed that MAPK4 is abundantly expressed in ECs of NSCLC and its expression is directly proportional to tumor angiogenesis.

### 3.2 MAPK4 silencing inhibits the proliferation of ECs in vitro

**Figure 2:**
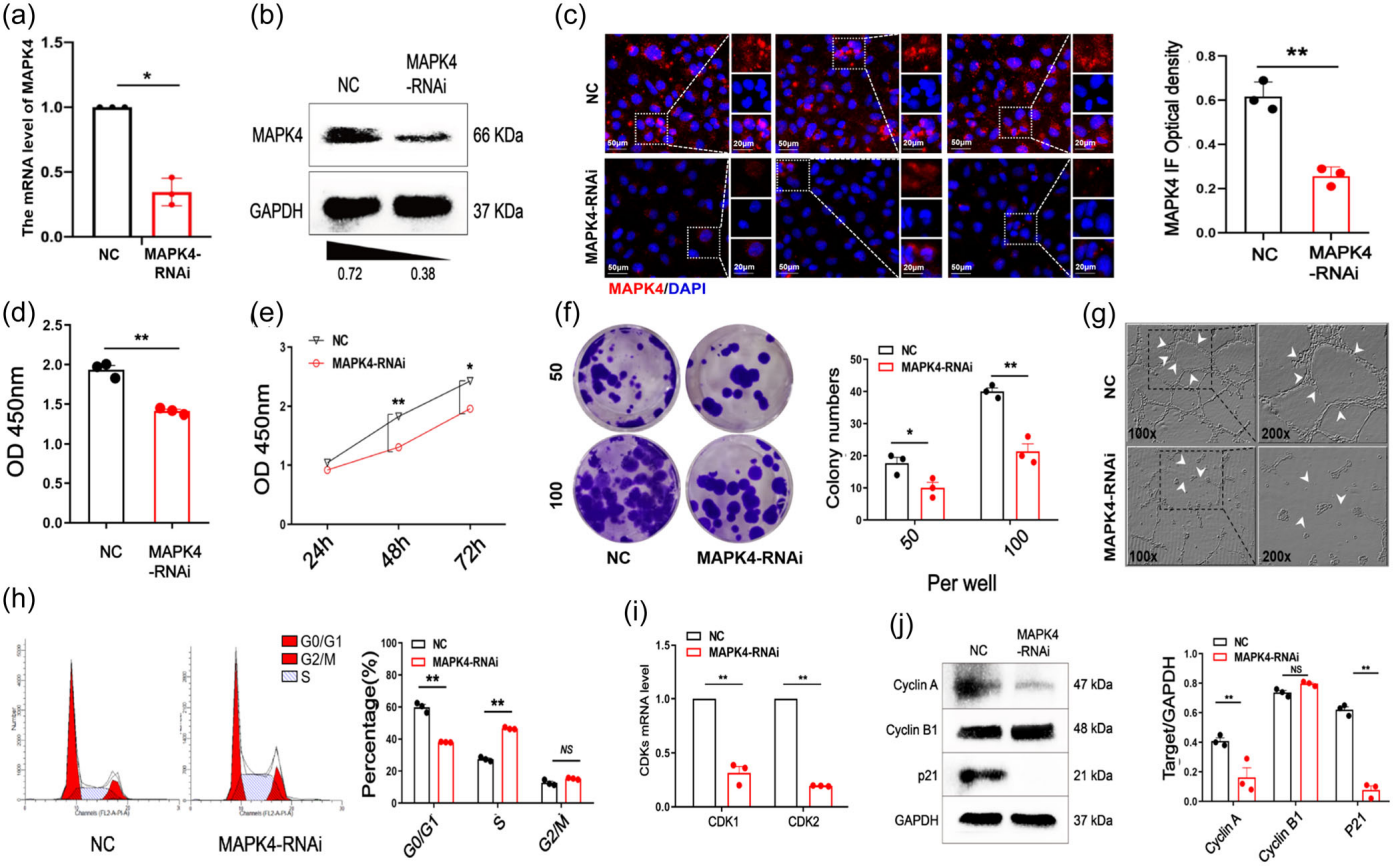
Mitogen‐activated protein kinase 4 (MAPK4) silencing inhibits the proliferarion of endothelial cells (ECs) in vitro. Human umbilical vein ECs (HUVECs) were transiently transfected with MAPK4 small interfering RNA (siRNA) (50 nM) in 24‐well plates via Lipofectamine 3000 reagent in vitro, 48 h later. (a) The expression of MAPK4 was analyzed by real‐time polymerase chain reaction (PCR). (b) Western blot analysis and (c) immunofluorescence staining were used to detect and quantitatively analyze MAPK4 expression in the two groups. (d, e) Cell counting kit‐8 (CCK‐8) assay was used to evaluate the proliferation of HUVECs 24, 48, and 72 h after transfection of MAPK4‐RNA interference (RNAi). (f) Colony formation assay was used to evaluate the colony‐forming ability of HUVECs and the colonies were counted. (g) The tube‐forming ability of HUVECs was evaluated by tube formation assay. (h) The cell cycle distribution was evaluated by fluorescence‐activated cell sorting. (i) The expression levels of CDKs in HUVECs were determined by real‐time PCR. (j) Western blot analysis was used to evaluate the expression of Cyclin A, Cyclin B1, and cyclin‐dependent kinase inhibitor 1A (p21) in HUVECs. Representative data from three independent experiments were shown. *p < 0.05, **p < 0.01. NS, no significance.

Our results showed that MAPK4 was highly expressed in ECs in NSCLC tumors. We thus next explored whether MAPK4 contributes to angiogenesis in NSCLC. Because of the limitation of obtaining primary ECs in tumors, we used HUVECs. We then examined MAPK4 function using transiently transfected MAPK4 RNA interference (RNAi). Quantitative real‐time PCR, western blot analysis, and immunofluorescence results showed that the expression of MAPK4 in MAPK4 RNAi‐transfected HUVECs was markedly lower than that in the control group (Figure 2a–c). CCK‐8 assay results indicated that the proliferation ability of MAPK4 RNAi‐transfected HUVECs was markedly decreased (Figure 2d) in a time‐dependent manner (Figure 2e). Moreover, the colony‐forming and tube‐forming abilities of MAPK4 RNAi‐transfected cells were decreased (Figure 2f,g). Notably, scratch assay results showed that the migration ability of MAPK4 RNAi‐transfected cells was unchanged compared with the control group (Supporting Information S1: Figure S2A–E).

We further examined the influence of MAPK4 on cell cycle distribution. FCM results showed that the percentage of G0/G1‐phase cells in the MAPK4 RNAi‐transfected group was markedly lower than that in the control group, whereas the percentage of S‐phase cells was increased (Figure 2h), indicating that MAPK4 silencing induces S phase arrest. We next examined the expressions of cell cycle‐related proteins. Real‐time PCR results showed that the mRNA levels of cyclin‐dependent kinases (CDKs) were lower in the MAPK4 RNAi‐transfected group than in the control group (Figure 2i). Western blot analysis results showed that cyclin A and p21 expressions in the MAPK4 RNAi‐transfected group were lower than those in the control group (Figure 2j), but the level of cyclin B1 was not significantly different (Figure 2j). These results suggested that MAPK4 silencing inhibited cell proliferation and induced S phase arrest of ECs.

### 3.3 MAPK4 silencing inhibits the growth of HUVECs by regulating the Raf/MEK/ERK1/2 signaling pathway

**Figure 3:**
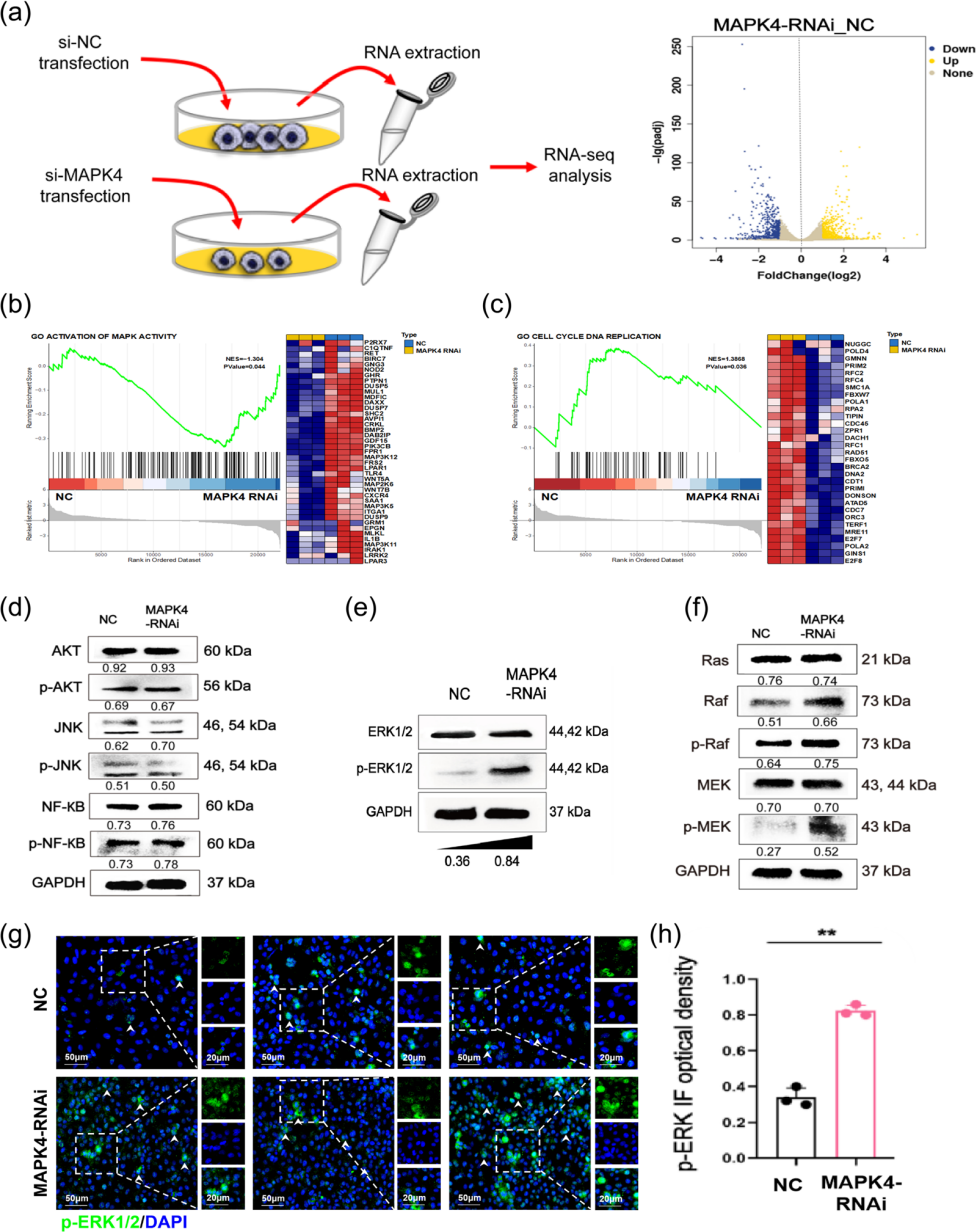
Mitogen‐activated protein kinase 4 (MAPK4) silencing inhibits the proliferation of endothelial cells (ECs) by regulating the Raf/MEK/ERK1/2 signaling pathway. Human umbilical vein ECs (HUVECs) were transiently transfected with MAPK4 small interfering RNA (siRNA) (50 nM) in 24‐well plates via Lipofectamine 3000 reagent in vitro. (a) Volcano plot showing genes with differential expression in MAPK4‐silenced HUVECs (MAPK4 HUVECs) compared with NC HUVECs, as determined by RNA sequencing (RNA‐seq). *n* =  3 per group. (b, c) Gene set enrichment analysis plots (left) and heat maps (right) of the RNA‐seq data for NC HUVECs and MAPK4 HUVECs. (d) Western blot analysis was used to evaluate the levels of protein kinase B (AKT), phosphorylated AKT (p‐AKT), c‐Jun n‐terminal kinase (JNK), phosphorylated JNK (p‐JNK), nuclear factor κB (NF‐κB), phosphorylated NF‐κB (p‐NF‐κB), (e) ERK1/2, phosphorylated ERK1/2 (p‐ERK1/2), (f) rat sarcoma (Ras), Raf, p‐Raf, MEK, and phosphorylated MEK (p‐MEK) in HUVECs. (g) 24 h after transfection, transfected cells were treated with the p‐ERK1/2 inhibitor and cultured for another 24 h. (h) Immunofluorescence was used to evaluate and quantitatively analyze the p‐ERK1/2 level in HUVECs. Representative data from three independent experiments are shown. **p < 0.01. ERK1/2, extracellular regulated protein kinases 1/2; MEK, mitogen‐activated extracellular signal‐regulated kinase; Raf, rapidly accelerated fibrosarcoma.

To further investigate the role and mechanism of MAPK4 in angiogenesis, we used global gene expression analysis to evaluate gene expression changes after MAPK4 silencing. RNA sequencing revealed that 542 genes were upregulated and 608 genes were downregulated in the MAPK4 RNAi‐transfected group (Figure 3a). Gene Ontology analysis showed that MAPK‐related genes were significantly upregulated (Figure 3b). In line with in vitro experiments, cell cycle‐related genes and DNA replication‐related genes were significantly downregulated in the MAPK4 RNAi‐transfected group (Figure 3c). These data suggested that MAPK4 silencing influences pathways involved in proliferation and the cell cycle in ECs.

Our above data showed that MAPK4 silencing inhibited the growth of ECs and influences multiple intracellular signaling pathways including other MAPKs pathways. We thus further examined potential changes in these signaling pathways. Western blot analysis results showed that ERK1/2, AKT, p‐AKT, NF‐κB, p‐NF‐κB, JNK, and p‐JNK levels were not significantly different in the MAPK4 siRNA‐transfected and control groups (Figure 3d,e). However, p‐ERK1/2 was markedly increased in the MAPK4 siRNA‐transfected group (Figure 3e). Therefore, we speculated that MAPK4 silencing may lead to activation of ERK1/2, but not AKT, JNK, and NF‐κB signaling pathways in ECs.

To further examine changes in the ERK1/2 pathway, a three‐kinase module, we used western blot analysis to evaluate MEK (MAPKK) and Raf (MAPKKK), and their phosphorylated forms. The levels of p‐MEK, Raf, and p‐Raf in the MAPK4 siRNA‐transfected group were markedly increased compared with levels in the control group (Figure 3f). To further confirm the effect of MAPK silencing on the ERK1/2 pathway, we used immunofluorescence to examine activation of the ERK1/2 pathway in MAPK4 RNAi‐transfected HUVECs in vitro by immunofluorescence. The results confirmed that p‐ERK1/2 expression in the MAPK4 RNAi‐transfected group was increased compared with controls (Figure 3g,h).

**Figure 4:**
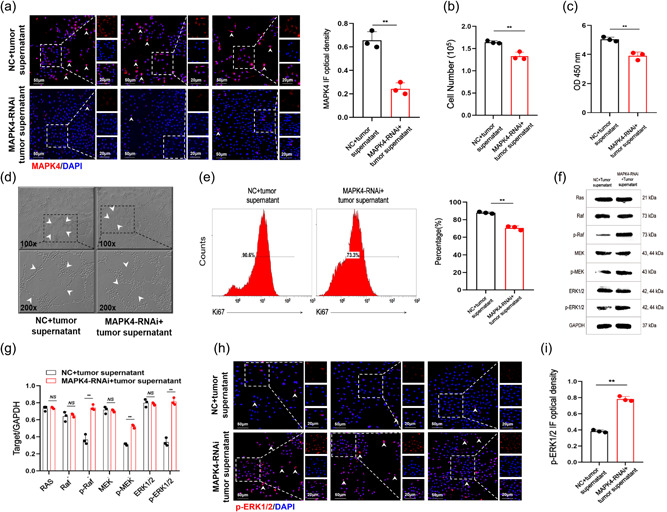
Mitogen‐activated protein kinase 4 (MAPK4) silencing inhibits endothelial cell (EC) growth. Human umbilical vein ECs (HUVECs) were cultured in the supernatant of human non‐small cell lung cancer (NSCLC) cells and transiently transfected with MAPK4 small interfering RNA (siRNA) (50 nM) in 24‐well plates via Lipofectamine 3000 reagent in vitro. (a) The expression of MAPK4 in HUVECs was detected by immunofluorescence. (b) The number of cells was determined by cell counting. (c) Cell counting kit‐8 (CCK‐8) assay was used to evaluate the proliferation ability of HUVECs. (d) The tube‐forming ability of HUVECs was evaluated by tube formation assay. (e) The expression of ki‐67 in HUVECS was detected by fluorescence‐activated cell sorting. (f, g) Extracellular regulated protein kinases 1/2 (ERK1/2) and phosphorylated ERK1/2 (p‐ERK1/2) in HUVECs was detected by western blot analysis. (h, i) The expression of p‐ERK1/2 in HUVECs was detected by immunofluorescence analysis and calculated. Representative data from three independent experiments are shown. **p < 0.01, NS, no significance.

To reproduce the tumor microenvironment in vivo, we cultured HUVECs in the supernatant of human NSCLC A549 cells and examined changes of growth in HUVECs after MAPK4 silencing. Immunofluorescence results confirmed that MAPK4 expression was significantly lower in the MAPK4 RNAi‐transfected group compared with the control group (Figure 4a). Consistent with the above data, cell counting, CCK‐8 assay, tube formation assay, and FCM results showed that the proliferation and tube‐forming abilities of HUVECs were decreased in the MAPK4 RNAi‐transfected group (Figure 4b–e). Similar results were obtained in HUVECs cultured in the supernatant of A549 cells. The expressions of p‐Raf, p‐MEK, and p‐ERK1/2 were increased in the MAPK4 RNAi‐transfected group, while ERK1/2, MEK, Raf, and Ras levels were not significantly different (Figure 4f,g). We further examined p‐ERK1/2 expression by immunofluorescence and obtained similar results (Figure 4h,i). Together, these data indicated that the effects of MAPK4 silencing in the growth of HUVECs are related to changes in the transduction of the Raf/MEK/ERK1/2 pathway.

### 3.4 High expression of MAPK4/CD34 is associated with poor survival of patients with NSCLC 

**Figure 5:**
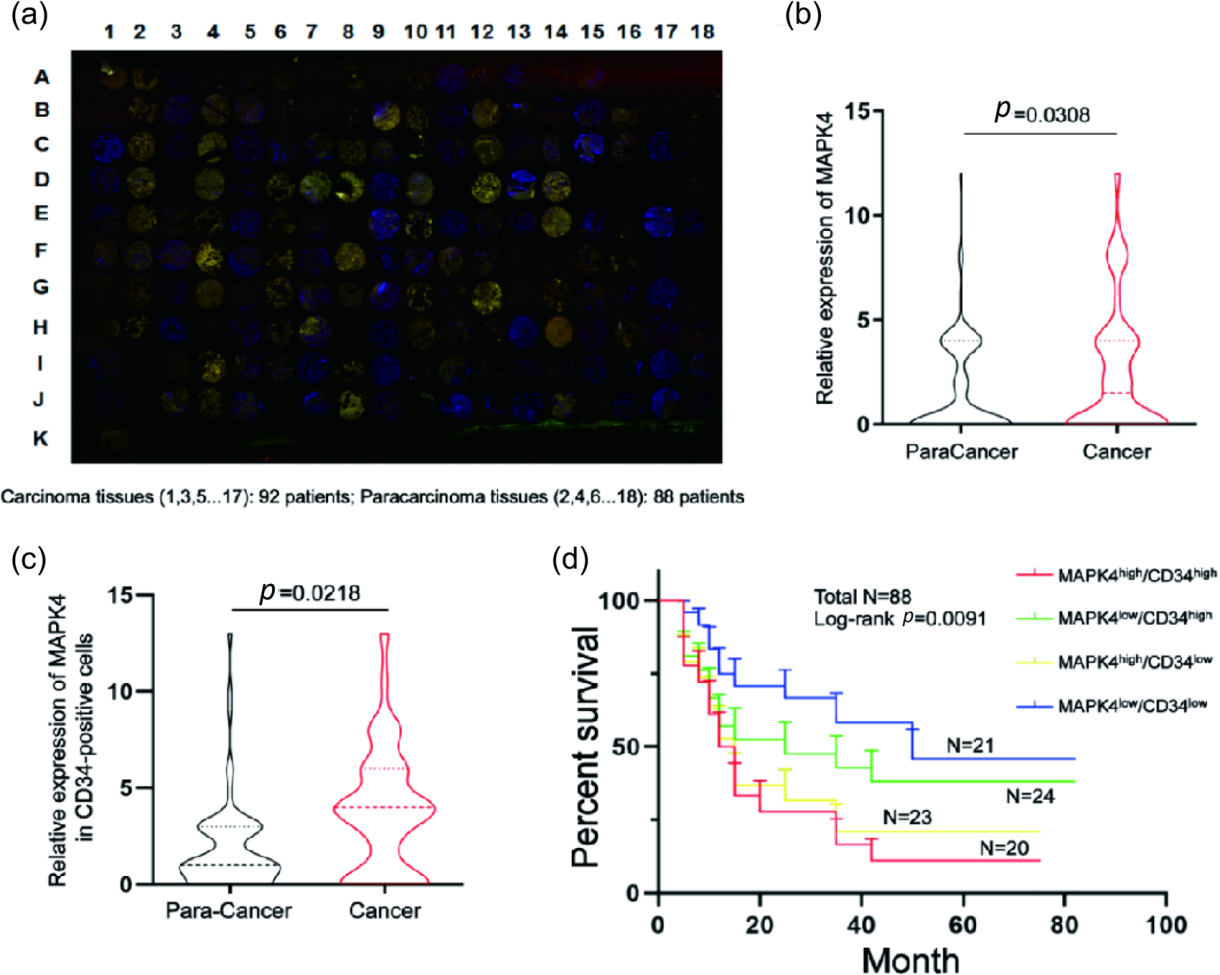
The expression of mitogen‐activated protein kinase 4 (MAPK4) in the tumor tissue of patients with clinical non‐small cell lung cancer (NSCLC). (a) The microarray of NSCLC tissues obtained from relevant patients was analyzed by multiplex immunofluorescence staining of CD34 and MAPK4. MAPK4 staining is shown in red, CD34 staining is shown in green, and 4′,6‐diamidino‐2‐phenylindole (DAPI) staining is shown in blue. Carcinoma tissues (1, 3, 5…17): 92 samples; paracarcinoma tissues (2, 4, 6…18): 88 samples. (b) The expression of MAPK4 in carcinoma and paracarcinoma clinical samples from patients with NSCLC were analyzed, and the H‐scores were calculated. (c) Comparison of MAPK4 expression in CD34‐positive cells between cancer and adjacent tissues. (d) The 5‐year overall survival rate in the four groups was determined through Kaplan–Meier analysis.

We found that MAPK4 promotes the growth of NSCLC by regulating tumor angiogenesis. To verify the relationship between MAPK4 and angiogenesis in clinical samples, we performed tissue microarray‐based immunofluorescence analysis of MAPK4 expression and examined the relationship between MAPK4 and CD34 in NSCLC samples (Figure 5a). In line with previous work [19], our results showed that MAPK4 expression in cancer tissues was markedly higher than that in adjacent tissues (Figure 5b). However, there was no correlation between MAPK4 expression and sex, age, or other clinical characteristics (Table 1). Moreover, the expression level of MAPK4 in CD34‐positive ECs in cancer tissues was significantly higher than that in adjacent tissues (Figure 5c). Furthermore, patients with NSCLC with high MAPK4/CD34 showed a shorter survival rate (Figure 5d). These results suggested that MAPK4 is highly expressed in cancer tissues of patients with NSCLC and, the prognosis of patients with NSCLC with MAPK4^high^/CD34^high^ tumors is poor.

**Table 1:** Correlations of MAPK4 expression with clinical characteristics of patients with NSCLC.

Characteristics	Total	MAPK4 in cancer		*χ* ^2^	*p*
		Low	High		
Age (years)					
≥60	55	25	30	1.212	0.271
<60	33	19	14		
Sex					
Male	49	22	27	1.151	0.283
Female	39	22	17		
Histological grade					
I/II	56	28	28	0.000	1.000
III	32	16	16		
T					
T1–2	57	26	31	0.281	0.596
T3–4	19	10	9		
N					
N0	34	14	20	0.720	0.396
N1–3	33	17	16		
Tumor size					
≥5	19	10	9	0.281	0.596
<5	57	26	31		
Clinical stage					
I–II	39	17	22	0.433	0.511
III–IV	25	13	12		
ALK					
Negative	72	35	37	0.446	0.307
Positive	9	6	3		
EGFR					
Negative	67	31	36	1.564	0.211
Positive	21	13	8		

Abbreviations: ALK, anaplastic lymphoma kinase; CDK, cyclin‐dependent kinase; EGFR, epidermal growth factor receptor; MAPK4, mitogen‐activated protein kinase 4; NSCLC, non‐small cell lung cancer.

### 3.5 Targeted silencing of MAPK4 in ECs inhibits the growth of NSCLC in vivo

**Figure 6:**
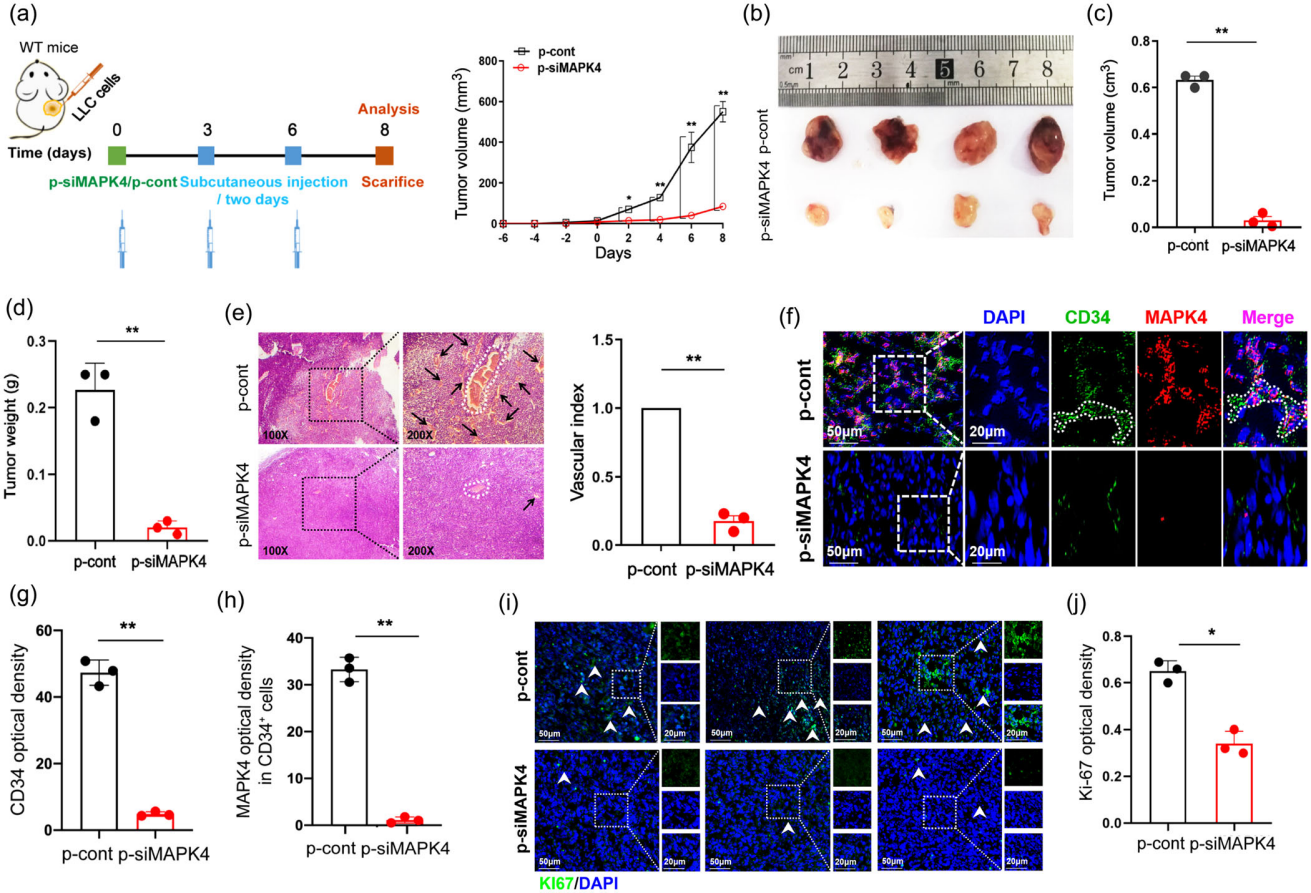
Targeted intervention of the expression of mitogen‐activated protein kinase 4 (MAPK4) could inhibit the growth of non‐small cell lung cancer (NSCLC) tumor in vivo. Lewis lung carcinoma (LLC) cells (5 × 10^5^) were subcutaneously injected into the right flanks of wild‐type (WT) C57BL/6 mice. Tumors were collected on day 21. (a) Schematic diagram showing subcutaneously injection of LLC cells (5 × 10^5^) into the right flanks of WT mice. Seven days later, the constructed PGL3.0 basic‐CD34 promoter‐MAPK4 RNA interference (RNAi) eukaryotic expression vectors (termed as p‐siMAPK4) or p‐cont given by subcutaneous injection into the left flank of murine NSCLC tumor model three times every 3 days. Tumor growth was monitored every 2 days to generate the growth curve, and tumors were collected on day 14. (b) Tumor size. (c) Tumor volume and (d) tumor weight. (e) Tumor pathology was analyzed by hematoxylin‐eosin (H&E) staining. The arrows indicate neovascularization. (f–h) The expression of MAPK4 and CD34 in tumors was analyzed by immunofluorescence and quantitated. (i, j) Cell proliferation nuclear antigen (Ki67) expression in tumors was analyzed by immunofluorescence and quantitated. Representative data from three independent experiments were shown. *p < 0.05, **p < 0.01.

As the expression of MAPK4 was proportional to tumor angiogenesis and MAPK4 silencing inhibited the growth and migration ability of ECs, we then further investigated the potential value of MAPK4 as an antiangiogenesis target in NSCLC. The data showed that the tumor growth was slower in the p‐siMAPK4 intervention group (Figure 6a). Additionally, tumor volumes were markedly smaller, and tumor weights were markedly lower in the p‐siMAPK4 intervention group (Figure 6b–d). H&E staining revealed that local vascular density of tumors in the p‐siMAPK4 group was decreased (Figure 6e). The levels of CD34^+^ ECs in tumors were also reduced (Figure 6f,g). Notably, MAPK4 expression was markedly decreased in CD34^+^ ECs but not in NSCLC cells (Figure 6f,h). Furthermore, Ki67 expression in tumors was lower in the p‐siMAPK4 intervention group (Figure 6i,j). Therefore, these data suggested that targeted silencing of MAPK4 in ECs effectively inhibited local angiogenesis and subsequently reduced the growth of NSCLC.

Next, we observed the distributions of p‐siMAPK4 plasmids in several organs and tissues to evaluate the potential influence of MAPK4‐targeting interventions in the body. The data showed that p‐siMAPK4 was dominantly enriched in vascularized tissues, such as lung, heart, and tumor tissues (Supporting Information S1: Figure S3A). However, MAPK4 expression was markedly downregulated in tumor tissues compared with other tissues (Supporting Information S1: Figure S3B). We also observed no significant changes in histology and weight in six organs and tissues, including heart, liver, spleen, lung, brain, and colon, between the groups (Supporting Information S1: Figures S3C–H and S4A). To confirm these findings, we examined the concentration of serum AST, ALT, and other key indicators for these organs and tissues. The results showed that these biochemistry indicators were not significantly changed between the groups (Supporting Information S1: Figure S4B–G). Together, these data suggested that MAPK4 may be a potential antiangiogenic target for NSCLC, and CD34 promoter‐operated siMAPK4 may be a valuable strategy for clinical intervention.

## 4 DISCUSSION

Our current study reveals that MAPK4 was abundantly expressed in ECs and its high expression positively correlated with tumor angiogenesis. MAPK4 silencing inhibited the proliferation and migration abilities of HUVECs in vitro. Mechanistically, MAPK4 silencing inhibited the proliferation and migration abilities of HUVECs by affecting the ERK1/2 pathway, accompanied by changes in the expression of multiple genes associated with cell cycle and angiogenesis. Notably, the expression of MAPK4 in CD34‐positive cells was closely related with poor prognosis in patients with clinical NSCLC. Finally, targeted silencing of MAPK4 in ECs using siRNA driven by the CD34 promoter effectively inhibited the angiogenesis and growth of NSCLC in vivo.

The MAPK family is a family of serine/threonine kinases that play a central role in transducing extracellular signals to induce various reactions within the cells [28]. The MAPK family is divided into two categories: typical MAPKs and atypical MAPKs. Typical MAPKs include ERK1/2, JNK 1/2/3, p38 isoforms (α, β, γ, and δ), and ERK5 [29]. Atypical MAPKs include MAPK4, MAPK6, MAPK15, and NLK [17]. Accumulating evidence has supported an association between MAPKs, including MAPK4, and various types of malignant tumors, including prostate cancer [30], ovarian cancer [24], and NSCLC [31, 32]. MAPK4 promotes prostate cancer by concerted activation of androgen receptors and AKT [30]. miR‐127‐3p inhibits the proliferation of rat ovarian cancer cells by downregulating MAPK4 [24], and the overexpression of MAPK4 promotes the growth of NSCLC cell lines by activating the AKT/mTOR signaling pathway [27]. In the present study, we extended the previous findings by demonstrating that compared with other members of the atypical MAPK family, MAPK4 was highly expressed in ECs of NSCLC, and its expression was related to increased tumor vascular density. Notably, we found that targeted silencing of MAPK4 in ECs also inhibited angiogenesis and abrogated the tumorigenesis of NSCLC in vivo. Moreover, we analyzed clinical samples of patients with NSCLC and found that the expression of MAPK4 was higher in tumor tissues than that in adjacent tissues. CD34 is a representative marker of new vessels. Our results showed that the expression of MAPK4 in cells expressing CD34 was closely related to the poor prognosis of patients with NSCLC. Therefore, our data revealed a novel biological role for MAPK4 in angiogenesis during NSCLC tumorigenesis, which suggests a pivotal role for MAPK4 in the development of NSCLC.

ECs play a key role in neovascularization during angiogenesis. Many studies have reported that MAPKs are involved in the proliferation and growth of ECs under different conditions. For example, Li et al. [33] found that elevated p38 and ERK1/2 activity was associated with apoptosis of ECs. Wang et al. [34] reported that the low levels of H_2_O_2_ significantly promoted the proliferation, migration, and tube formation of ECs through the JNK pathway. Our results herein showed that MAPK4 silencing impaired the proliferation, colony‐forming and tube‐forming abilities of HUVECs in vitro, accompanied by cell cycle arrest and altered expression of cell cycle‐related genes.

To reproduce the tumor microenvironment in vitro, we cultured HUVECs in the supernatant of human NSCLC cells. Under these conditions, MAPK4 silencing also inhibited the growth of HUVECs in vitro, which was consistent with the experimental results in vivo. In line with these findings, global gene expression analysis showed that MAPK4 silencing resulted in changes in the expression patterns of cell cycle‐related genes. The mechanism by which MAPK4 silencing induces S‐phase arrest in HUVECs requires further study. Additionally, we found that MAPK4 silencing did not affect the migration ability of ECs. These results indicated that MAPK4 plays an important role in the growth of ECs, thereby promoting the angiogenesis of NSCLC. Recent studies have shown that interactions among multiple cells in the tumor microenvironment, including tumor cells, ECs, and immune cells, are important for tumor angiogenesis. Therefore, it may be valuable to further explore the potential influence of other cells in local tumor tissues on the expression of MAPK4 in ECs. This information may help aid in the identification of the exact biological function of MAPK4 in tumor angiogenesis and development of NSCLC.

Multiple signaling pathways, such as the MAPK, AKT, and NF‐κB pathways, are connected by complex networks in eukaryotic cells, including ECs, and control gene expression, cell survival, apoptosis, and cell differentiation [35, 36, 37, 38]. Notably, our results showed that MAPK4 silencing did not affect the AKT, JNK, and NF‐κB signaling pathways in ECs. However, signal transduction through the Raf/MEK/ERK1/2 pathway was significantly increased in the absence of MAPK4 and in HUVECs cultured in the supernatant of human NSCLC cells. Importantly, inhibition of ERK1/2 pathway reversed the effect of MAPK4 silencing on the growth and tube‐forming ability of ECs. The ERK1/2 pathway plays an important role in the growth of ECs. For example, Zong et al. [39] reported that Notch1 protects against cigarette smoke‐induced endothelial apoptosis in chronic obstructive pulmonary disease by inhibiting the ERK pathway. Taken together, these data indicated that the effect of MAPK4 silencing on the proliferation of HUVECs is related to the altered signal transduction through the Raf/MEK/ERK1/2 signaling pathway. Notably, Chen et al. [40] reported that administration of liraglutide promoted the activation of ERK and the survival of ECs, indicating that the ERK1/2 pathway also might play distinct roles in the growth of ECs under different conditions. Moreover, in our previous study, we found that MAPK4 deficiency altered signal transduction through the NF‐κB and JNK pathways in ALI [18]. We speculate that the results of these studies reflect the complexity of the exact roles of the networks connecting these pathways in different experimental settings. Thus, the precise interaction between MAPK4 and the Raf/MEK/ERK1/2 pathway in the tumor angiogenesis and development of NSCLC needs to be fully elucidated.

## 5 CONCLUSION

**Figure 7:**
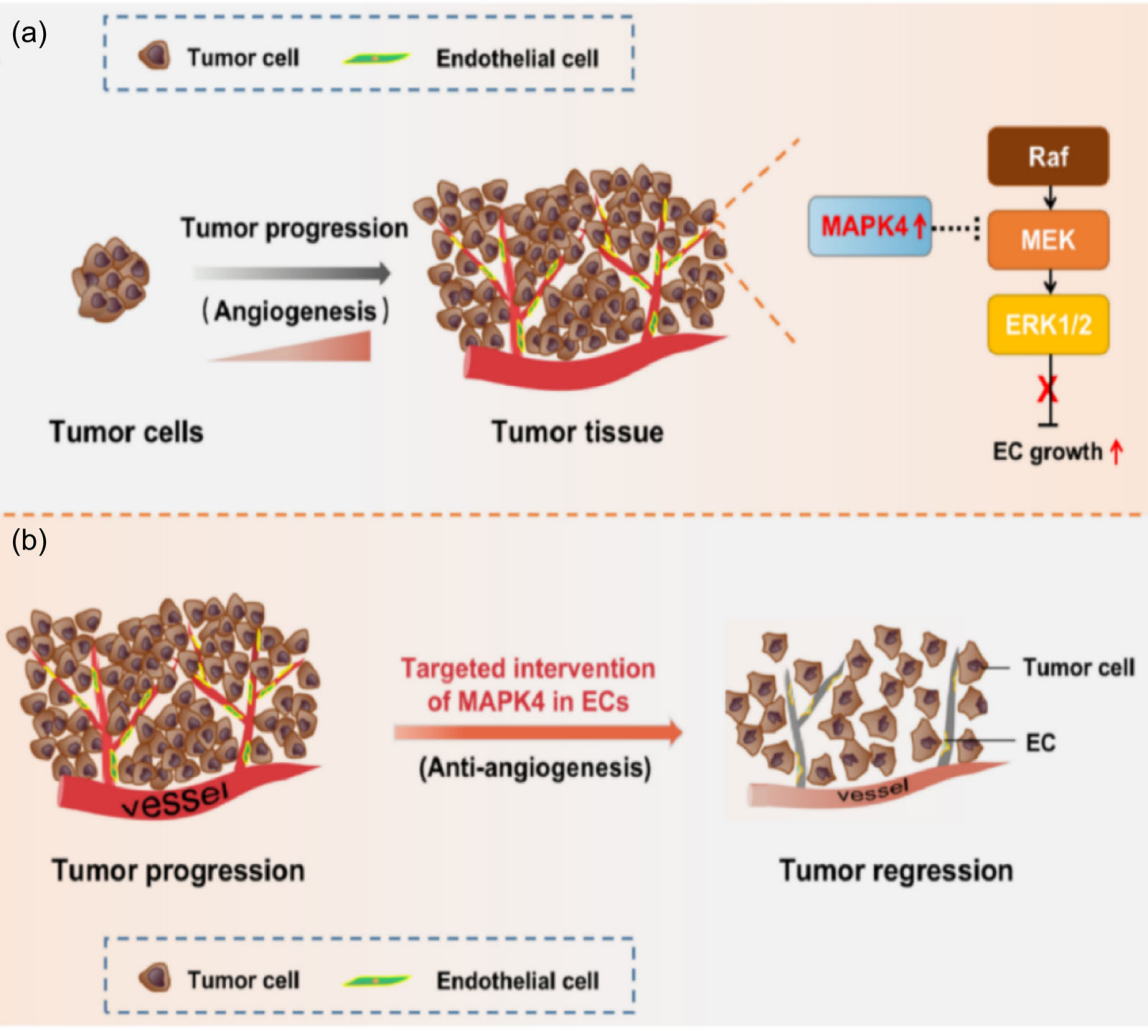
Schematic diagram of mitogen‐activated protein kinase 4 (MAPK4) role in tumor angiogenesis and progression of non‐small cell lung cancer (NSCLC). (a) MAPK4 facilitates tumor angiogenesis and progression in NSCLC by promoting the growth of endothelial cell (CD34^+^ EC), which is related to altered transduction of Raf/MEK/ERK1/2 signaling pathway. (b) Targeted intervention the expression of MAPK4 in CD34^+^ ECs can repress tumor angiogenesis and progression of NSCLC. ERK1/2, extracellular regulated protein kinases 1/2; MEK, mitogen‐activated extracellular signal‐regulated kinase; Raf, rapidly accelerated fibrosarcoma.

Our study revealed a previously unknown role for the atypical MAPK member MAPK4 in the angiogenesis and development of NSCLC. Our findings indicate that MAPK4 regulates the growth of ECs and accelerates angiogenesis through the ERK1/2 pathway, thus promoting the progression of NSCLC (Figure 7a). Furthermore, targeted intervention of MAPK4 expression in ECs might be a valuable clinical strategy to inhibit angiogenesis and induce tumor regression (Figure 7b). These findings indicate that MAPK4 may be a new clinical therapeutic target for NSCLC.

## AUTHOR CONTRIBUTIONS

Jing Chen, Jing Yang, and Yufang Liu designed the research. Jing Chen, Jing Yang, Yufang Liu, Juanjuan Zhao, Lin Tang, Xu Zhao, and Zhenke Wen conducted experiments. Jing Chen, Jing Yang, Yufang Liu, Ya Zhou, Chao Chen, Dongmei Li, and Mengmeng Guo analyzed data. Jing Chen, Jing Yang, and Yufang Liu wrote and revised this manuscript. Lin Xu and Guiyou Liang revised the manuscript and supervised the research. All authors read and approved the final manuscript.

## CONFLICT OF INTEREST STATEMENT

The authors declare no conflict of interest.

## ETHICS STATEMENT

Animal experiments were approved by and followed the ethical guidelines of the Zunyi Medical University Laboratory Animal Care and Use Committee, China (No. 2018016). The data extraction and tissue microarray construction of patients with lung cancer were approved by the ethics committee of Shanghai Outdo Biotech Co., Ltd. All authors of this study have seen and approved the current version of the manuscript.

## INFORMED CONSENT

The authors have nothing to report.

## Supporting information

Supporting Information

## Data Availability

The data sets in this study are available from the corresponding author on reasonable request.
