# Supplementary material for: MAPK4 facilitates angiogenesis by inhibiting the ERK pathway in non‐small cell lung cancer
Source: Cancer Innov. 2024 Apr 16;3(3):e117. doi: 10.1002/cai2.117 (PMC11212285; doi:10.1002/cai2.117)
Supplement: Supplementary file 1 — Supporting Information [file CAI2-3-e117-s001.docx]

**[Supplemental I](https://www.elsevier.com/journals/acta-pharmaceutica-sinica-b/2211-3835/guide-for-authors#txt87000)nformation**

**MAPK4 facilitates angiogenesis by inhibiting the ERK pathway in non-small cell lung cancer**

Jing Chen^1,2,#^, Jing Yang^1,2,#^, Yufang Liu^1,2,#^, Xu Zhao^1,2^, Juanjuan Zhao^1,2^, Lin Tang^1, 2^, Mengmeng Guo^1,2^, Ya Zhou ^1,3^, Chao Chen^1,2^, Dongmei Li^1,2^, Zhenke Wen^4^, Guiyou Liang^5,*^, Lin Xu^1, 2,*^

^1^ Special Key Laboratory of Gene Detection & Therapy of Guizhou Province, Guizhou, 563000, China

^2^ Department of Immunology, Zunyi Medical University, Guizhou, 563000, China

^3^ Department of Medical Physics, Zunyi Medical University, Guizhou, 563000, China

^4^ Institute of Biomedical, Soochow University, Jiangsu, 215006, China

^5^ Department of Cardiovascular Surgery, Affiliated Hospital of Guizhou Medical University, Guizhou, 563000, China

^#^These authors contributed equally to this work

Running title: MAPK4 facilitates angiogenesis in NSCLC.

**KEYWORDS**

NSCLC, MAPK4, p-ERK1/2, Angiogenesis, Endothelial cell

**^*^ Correspondence to:**

Dr. Lin Xu, Department of Immunology, Zunyi Medical University, Zunyi, 563003, Guizhou, China, Email: xulinzhouya@163.com or xulinzhouya@zmu.edu.cn

Dr. Guiyou Liang，Department of Cardiovascular Surgery, Affiliated Hospital of Guizhou Medical University, Guiyang 550004, Guizhou, China, Email: liangguiyou@gmc.edu.cn

**
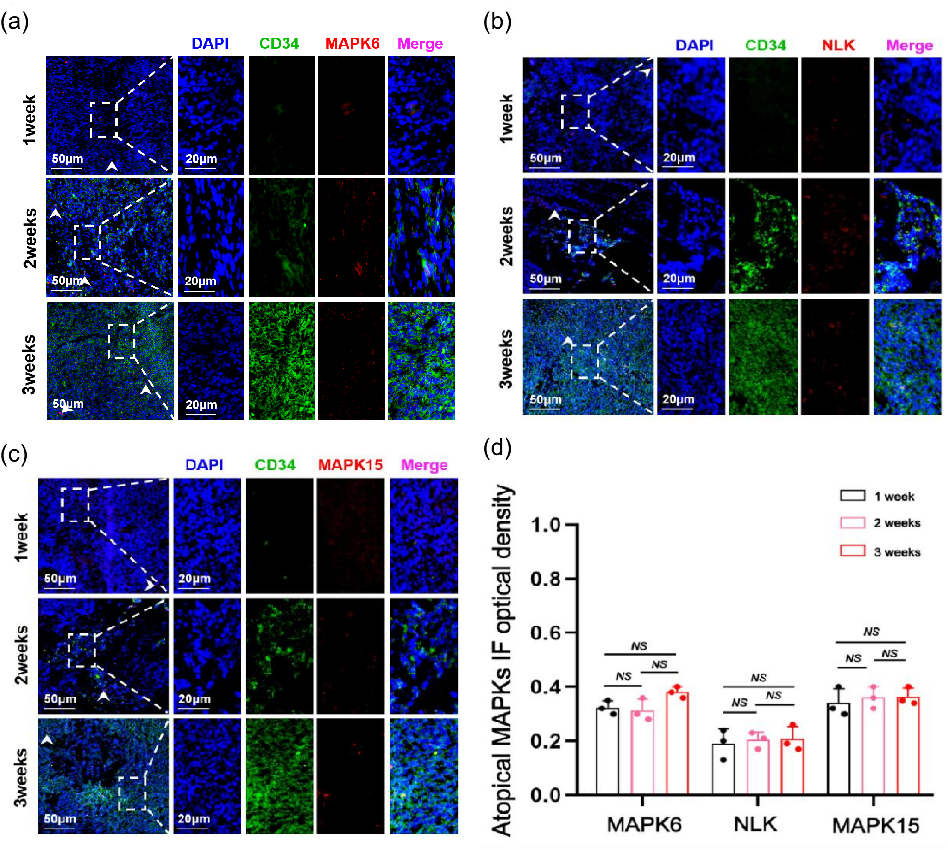

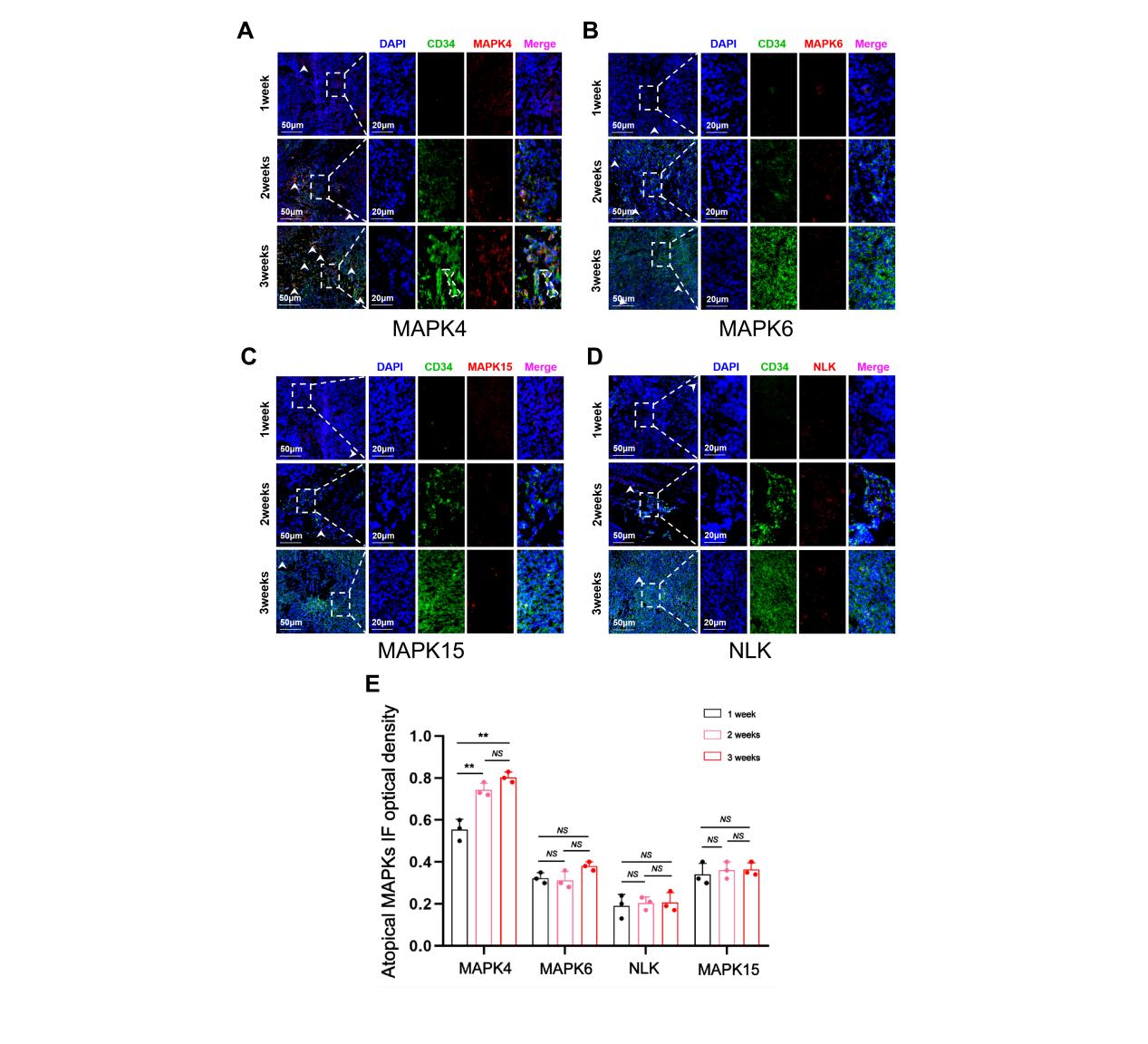
**

**Supplementary Figure 1. The expression level of MAPK6, NLK and MAPK15 in CD34^+^ ECs in tumor tissues.**

a-c) The expression levels of MAPK6, NLK and MAPK15 in tumors were analyzed by immunofluorescence and d) quantitated. Representative data from three independent experiments were shown. *NS*, no significance.

**
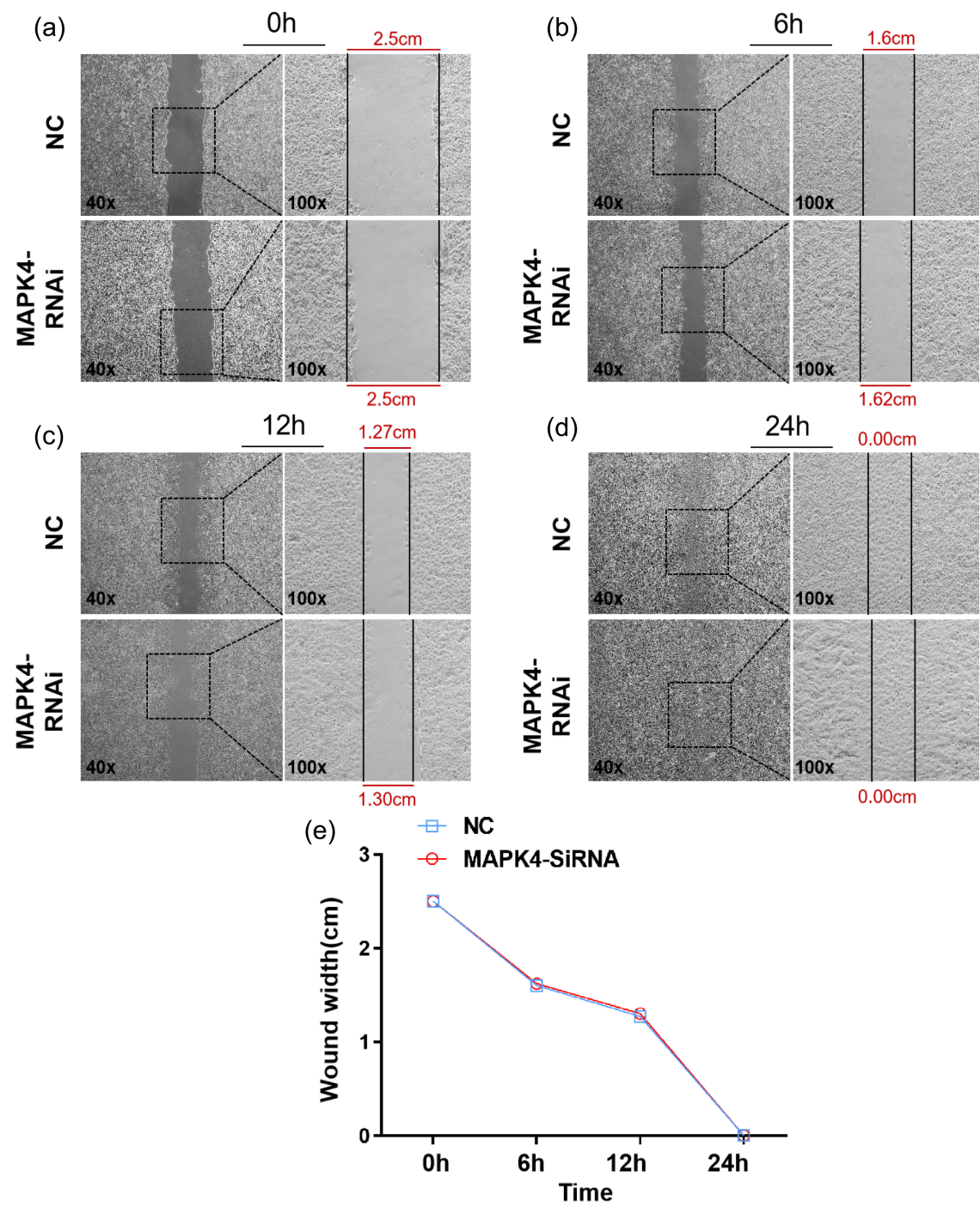
**

### **Supplementary Figure 2. The effect of MAPK4 silencing on migration of endothelial cell *in vitro*.**

### HUVECs were cultured in the supernatant of human NSCLC cells. HUVECs were transiently transfected with MAPK4 siRNA (50nM) in 24-well plates via Lipofectamine 3000 reagent *in vitro*. a-d) The effects of MAPK4-siRNA transfection on wound healing of HUVECs at 0, 6, 12 and 24h. e) The wound width was calculated at different time points. Representative data from three independent experiments are shown.

**
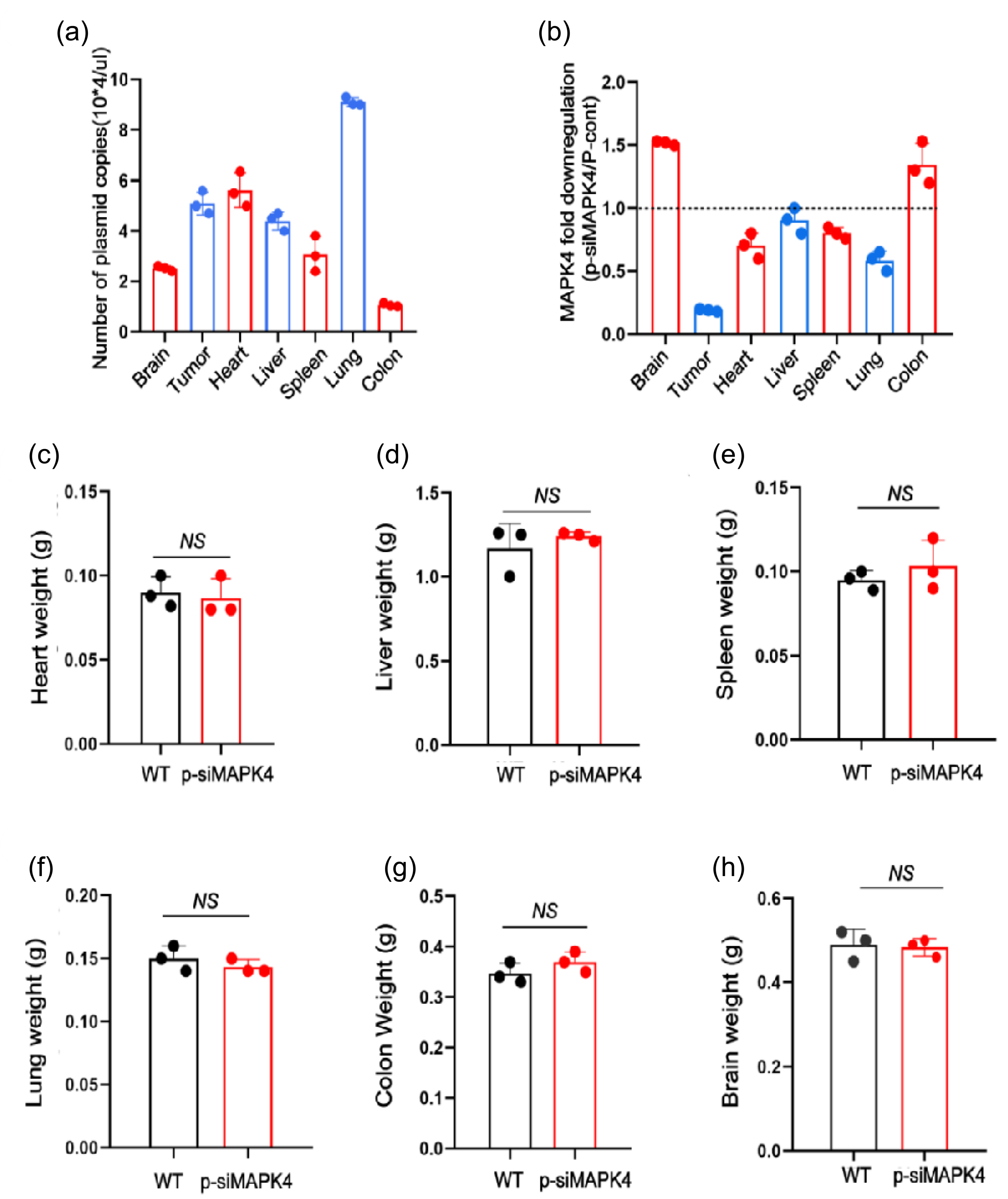
Supplementary Figure 3. The distribution of MAPK4 RNAi eukaryotic expression vector *in***

***vivo*.**

LLC cells (5×10^5^) were subcutaneously injected into the right flanks of WT mice. Seven days later, the plasmid of p-siMAPK4 or p-cont was remotely given by subcutaneous injection into the left flank of murine NSCLC tumor model three times every three days. 2 days after last injection, all major organs, including the brain, tumor, heart, liver, spleen, lung and colon, were harvested on day 14. a**)** DNA was purified and used for the calculation of plasmid copy number by real-time PCR. b**)** MAPK4 expression was measured by Realtime PCR assay in indicated organs. c**-**h**)** The weights of indicated organs were determined. Representative data from three independent experiments are shown. *NS,* no significance.

**
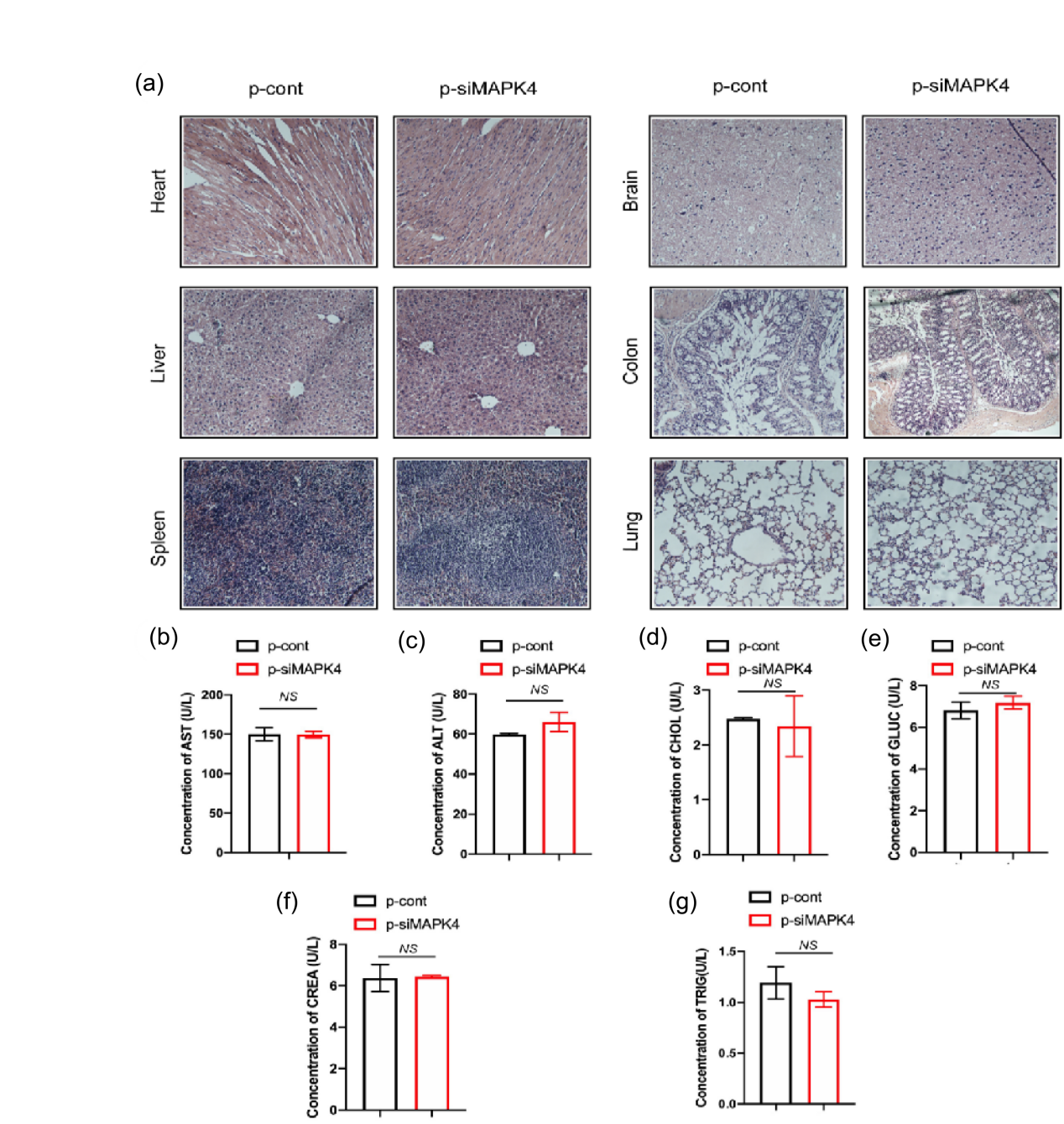
**

**Supplementary Figure 4. The change on important organs and tissues in murine NSCLC tumor model.**

LLC cells (5×10^5^) were subcutaneously injected into the right flanks of WT C57BL/6 mice. 7 days later, the plasmid of p-siMAPK4 or p-cont given by subcutaneous injection into the left flank of murine NSCLC tumor model three times every three days. 2 days after last injection, all of the major organs were obtained. a**)** The H&E staining of various organs and tissues, including heart, liver, spleen, lung, brain and colon, were performed. b**-**g**)** The concentration of the serum AST, ALT, CHOL, GLUC, CREA and TRIG were measured, respectively. *NS*, no significance.
